# How Can Implementation Science Improve the Care of Familial Hypercholesterolaemia?

**DOI:** 10.1007/s11883-023-01090-6

**Published:** 2023-02-20

**Authors:** Mitchell Sarkies, Laney K. Jones, Jing Pang, David Sullivan, Gerald F Watts

**Affiliations:** 1 School of Health Sciences, Faculty of Medicine and Health, University of Sydney, Sydney, NSW 2006 Australia; 2 Centre for Healthcare Resilience and Implementation Science, Australian Institute of Health Innovation, Faculty of Medicine, Health and Human Sciences, Macquarie University, Sydney, NSW Australia; 3 Department of Genomic Health, Research Institute, Geisinger, Danville, PA USA; 4 Heart and Vascular Institute, Geisinger, Danville, PA USA; 5 School of Medicine, University of Western Australia, Perth, WA Australia; 6 Department of Chemical Pathology, Royal Prince Alfred Hospital, Sydney, NSW Australia; 7 Lipid Disorders Clinic, Department of Cardiology, Royal Perth Hospital, Perth, WA Australia

**Keywords:** Implementation science, Familial hypercholesterolaemia, Detection, Statins, Clinical practice guidelines, Cholesterol

## Abstract

**Purpose of Review:**

Describe the application of implementation science to improve the detection and management of familial hypercholesterolaemia.

**Recent Findings:**

Gaps between evidence and practice, such as underutilization of genetic testing, family cascade testing, failure to achieve LDL-cholesterol goals and low levels of knowledge and awareness, have been identified through clinical registry analyses and clinician surveys. Implementation science theories, models and frameworks have been applied to assess barriers and enablers in the literature specific to local contextual factors (e.g. stages of life). The effect of implementation strategies to overcome these factors has been evaluated; for example, automated identification of individuals with FH or training and education to improve statin adherence. Clinical registries were identified as a key infrastructure to monitor, evaluate and sustain improvements in care.

**Summary:**

The expansion in evidence supporting the care of familial hypercholesterolaemia requires a similar expansion of efforts to translate new knowledge into clinical practice.

## Introduction

Familial hypercholesterolaemia (FH) is an inherited disorder of cholesterol metabolism, estimated to affect 1 in 250 of the general population [1, 2]. It is one of the most common autosomal dominant inherited genetic conditions, readily detectable through phenotypic and genetic testing [3, 4]. FH alters cholesterol metabolism from birth, resulting in elevated cholesterol levels and high risk for premature cardiovascular morbidity and mortality [2]. Early detection and treatment are clinically proven to cost-effectively prevent cardiovascular disease and improve survival rates [5•, 6–11]. This evidence base has informed international clinical practice guidelines which strongly recommend early detection, lifestyle modifications and pharmacotherapies to reduce low-density lipoprotein cholesterol (LDL-cholesterol) [5•, 6–8]. The management of FH is an exemplar of the implementation of precision medicine into routine clinical practice for the prevention of premature atherosclerotic cardiovascular disease (ASCVD) in individuals and families, owing to its relatively high prevalence and availability of effective preventative care.

The Centers for Disease Control and Prevention have created a 3-tier classification for genomic conditions for which evidence-based care is well supported and likely to have a major impact on health. FH has been identified as a tier 1 genomic application [12, 13], defined as having “sufficient evidence for clinical validity and utility to provide meaningful and actionable information to consumers and health care practitioners” [14, 15]. FH is more prevalent than other tier 1 genomic applications, such as hereditary breast and ovarian cancer and Lynch syndrome [12], and carries substantial potential for a positive impact on public health based on available evidence-based guidelines and recommendations [1, 2, 16]. Implementing public health programs to address FH provides a unique opportunity to apply complementary personalised and public-wide approaches to health care and disease prevention. Stratifying those with FH who are at risk of developing ASCVD could enable more efficient and effective prevention and management, potentially reducing the costs of care. Applying emerging methods for measuring disease risk and developing implementation strategies to improve health may help to reduce health disparities in populations [17] and the emerging concept of “genetic discrimination”.

### Evidence-to-Practice Gaps

Despite the clinical importance of FH, there are wide gaps between evidence-based guideline recommendations and routine clinical practice [18–21]. Less than 10% of people with FH have been detected worldwide [22]; and, of those who have been detected, only 20% attain guideline-recommended LDL-cholesterol goals [2, 12, 22, 23]. Although progress has been made to establish registries [24], global calls to action [25, 26], guidelines and position statements [6, 22, 27–31], efforts to reduce the burden of FH have been hampered by the lack of an integrated implementation strategy. This means most of the estimated 25 million people who have FH worldwide [2] remain at risk of developing ASCVD before 55 years of age in men and 60 in women (or before 20 for people with homozygous FH) [22].

Currently, people with FH tend to be diagnosed after the age of 40 years [24]. This delayed detection constrains the potential benefits of preventive treatment to reduce ASCVD risk. Furthermore, the opportunity for potential family-wide benefits is clear by averting premature death of relatives once a familial risk has been identified [32]. Improvements to FH detection are therefore necessary to identify individuals affected much earlier in their life course, as people identified at a younger age benefit from lower LDL-cholesterol and lower prevalence of cardiovascular risk factors and cardiovascular diseases [24]. Genetic testing is available in many countries; however, it is not widely implemented [33]. Optimal screening strategies have not yet been determined, as the respective roles of genetic testing, family history, and LDL-cholesterol testing require further examination in different country contexts.

Under-treatment of people with FH is common [33]. Evidence suggests 4 of 5 people receiving treatment are prescribed a single lipid-lowering medication, which without high-intensity therapy is unlikely to achieve guideline-recommended LDL-cholesterol concentrations [24]. Whilst greater use of combination therapy is indicated to improve FH management [24], barriers such as a general lack of awareness of FH among general practitioners, discomfort starting lipid-lowering treatment in younger patients, media misinformation and poor medication adherence exist [34]. Additionally, people with FH have indicated that improvements in screening and family-based care are needed [35].

The purpose of this review is to describe the application of implementation science to improve the detection and management of FH. We will review extant literature for each of the key steps in embedding implementation science into the guideline development and translation processes: (1) identifying evidence-to practice gap; (2) application of theories, models or frameworks; (3) assessing barriers and enablers to implementation; (4) tailoring implementation strategies; (5) monitor, evaluate and sustain improvements in care. We also discuss future directions for implementation research in FH.

## Identifying Evidence-to-Practice Gaps

The evidence-to-practice gap is a widely recognised phenomenon in health and medical research with some commentators suggesting it takes an average of 17 years for approximately 14% of all medical research to translate into practice [36]. FH is a condition that presents a relatively unique challenge for translating evidence into practice because it is predominantly asymptomatic for much of a person’s life, requiring preventative care. Identifying specific gaps in care where a substantial body of effectiveness and cost-effectiveness exists is an important first step to improving care provision [37]. However, these gaps in care will often need to be prioritised for the implementation of evidence-based recommendations when multiple competing areas are identified [38].

Gaps in the care of FH have been identified internationally, such as in Australia and the United States of America (USA), through the establishment of clinical registries and cross-sectional analysis of enrolled FH patients from lipid clinics [18, 23]. High proportions of the FH registry cohort have been identified as index cases, highlighting a substantial underutilisation of family cascade testing for the detection of FH. Specific to management, few patients achieved their LDL-cholesterol target goals. A similar study across 10 countries in the Asia-Pacific region and Southern Hemisphere used a series of questionnaires completed by key opinion leaders [21]. Overall, only 3% of the FH population were estimated to have been identified, which was perceived to be related to the amount of government expenditure on health care. Genetic testing and non-invasive imaging were infrequently used for detection and risk assessment. Approximately 30% of patients were thought to be achieving recommended LDL-cholesterol targets on statin therapies. Further treatment gaps included access to lipoprotein apheresis and proprotein convertase subtilsin-kexin type 9 (PCSK9) inhibitors. A deficit of FH registries, training programs, and publications was identified in low- and middle-income countries [21]. Health professional surveys have also uncovered gaps in knowledge and perceptions regarding the delivery of care for FH. In one survey, only around half of physicians were aware of the heritability of FH, and much fewer were familiar with the prevalence and severity of cardiovascular risk [19, 21].

## Application of Theories, Models or Frameworks

Implementation science theories, models and frameworks have been used primarily for three purposes: (1) describe the process of translating research into practice; (2) understand what factors (barriers and enablers) influence implementation success; and (3) evaluate implementation success [39]. The RE-AIM (Reach, Effectiveness, Adoption, Implementation and Maintenance) framework provides a valuable structure for describing and evaluating the process of implementation; it considers (1) the reach of those impacted by the implementation strategy, (2) the effectiveness of the strategy, (3) adoption of the strategy, (4) fidelity and costs of implementation and (5) maintenance of changes over time [40–42]. The Consolidated Framework for Implementation Research (CFIR) is widely used to assess barriers and enablers to implementation and categorise them according to the health system level/s impacted [43]. The authors of this review ran a series of implementation workshops on the detection of FH (Sarkies personal communication), where we categorised these factors according to the CFIR domains. Implementation strategies were matched to these factors, according to the Expert Recommendations for Implementing Change (ERIC) taxonomy (see Table 1 for examples) [44, 45].

**Table 1:** Example factors influencing the detection of FH in Australia categorised according to the CFIR domains and potential implementation strategies mapped to these factors

CFIR domain	Example influencing factors	Potential implementation strategies
Intervention characteristics	Lack of urgency for detecting and treating cholesterol	Conduct education meetings
Outer setting	Limited infrastructure for family genetic cascade testing	Access new funding
Inner setting	Prioritisation of FH detection in clinical practice	Identify and prepare clinical champions
Characteristics of individuals	Lack of awareness of FH	Conduct a local needs assessment
Process	Difficulties of procedures for obtaining family	Develop a formal implementation blueprint

## Assessing Barriers and Enablers to Implementation

Insufficient engagement with clinicians responsible for implementing guideline recommendations can lead to poor adoption. Often this is because strategies for implementation might not address the most salient barriers or enablers within local contexts [46]. A needs assessment of barriers and enablers is important to understand why gaps between evidence and practice exist before designing solutions. It is suggested that key relevant stakeholders are consulted, including those responsible for adopting, implementing, and sustaining changes in practice at different levels of the health system (e.g. patients, clinicians and policy makers) [47]. Several implementation frameworks have been developed to assess and categorise barriers and enablers to change, such as the CFIR [43]. Ideally, this should occur early in the implementation process, enabling the later selection and tailoring of strategies to address identified barriers and enablers for local contextual circumstances.

### FH Detection Barriers and Enablers

Hendricks-Sturrup et al. [48] conducted a systematic review in 2019 to identify barriers and facilitators to genetic testing for FH in the USA. They mapped 26 barriers and 15 enablers to FH genetic testing in the USA to the five CFIR domains: (1) characteristics of the intervention; (2) outer setting; (3) inner setting; (4) characteristics of individuals; (5) processes. The main factors related to the *intervention characteristics* were meeting diagnostic criteria, methods of DNA sample collection, costs and insurance coverage, availability of genetic counselling, testing result wait times, privacy and discrimination concerns, interpreting and using the test results and engagement of family members in cascade testing. Unique to the *outer setting* was access to testing services and the presence of expert consensus on genetic testing. The *inner setting* factors focussed on using electronic medical records (EMR) for detection, collaboration among clinicians, clarity of diagnostic criteria for EMR detection, time taken and accuracy of family history and adoption of tools for patients to conduct family history independently. *Individual characteristics* influencing genetic testing were patient-centred genetic counselling before and after genetic testing, patient readiness to undergo genetic testing, patient concerns and knowledge about FH, previous diagnosis of FH, patient use of educational materials and clinician perceptions of FH genetics within their scope of practice. Factors categorised as *processes* included clinician coordination and use of diagnostic codes, and use of FH phenotype-driven FH risk stratification and subsequent clinical management rather than genotype.

Specific to paediatric care, Wurtmann et al. [49] identified barriers and enablers to cascade screening in the USA using a survey questionnaire of 38 parents of children with FH. The most frequently reported enabler of living grandparent or aunt/uncle notification was to protect relatives from heart disease. Where notification did not occur, a lack of information about FH and the perceived ability of the relative to understand the information were highlighted as common barriers. Despite these concerns, less than half of survey respondents accessed educational institutional resources to share with relatives or assistance drafting a family notification letter.

### FH Management Barriers and Enablers

Barriers and enablers to the management of FH have been explored from both the clinician and patient perspectives. FH patients within the same family can have a different individual risk for ASCVD [50, 51], prompting some calls to re-stratify individual risk to improve lipid-lowering therapy [52, 53]. Recommended lipid-lowering targets can be difficult to achieve for some FH patients [54]. However, the price of higher-intensity lipid-lowering therapies such as PCSK9 inhibitors means availability has been limited to patients who will benefit most [55–57]. Patient support groups play an integral role in identifying barriers to accessing services and newer, expensive therapies [58, 59].

Jones et al. [60] conducted interviews and focus groups with 33 patients and 17 clinicians to evaluate stakeholder barriers and facilitators for the treatment of FH. Patients reported that medical professionals needed to be persistent with patients and families about the importance of treating FH. Having a great medical team with a good understanding of the condition and useful resources for FH were considered key enablers from the patient’s perspective. Patients also mentioned several barriers: changing guidelines; gaps in care provision; non-disclosure of family history; a lack of awareness of treatments and insurance coverage (including loss of employment and associated insurance); reluctance to take treatments due to side effects; and competing personal life demands all got in the way of FH care. Clinicians considered having knowledge of treatment options, genetic test results and the availability of clear diagnostic criteria as key enablers to good FH care. Several barriers were identified at the clinician level: lack of awareness of FH and treatment options; perceived lack of evidence to support some treatments; challenges convincing patients to adhere to treatments; incompatibility of medical records; and other competing priorities.

Patient and family lived experience perspectives have now been mapped to the priorities outlined in the 2020 FH Global Call to Action [25, 35]. Patients reported whilst some family members were receptive to information; others avoided or reacted negatively to information about FH. For those with receptive family members, family appointments with health professionals could enable immediate screening and care planning for the whole family. These appointments were particularly important for those who did not usually discuss health-related problems within their family units. People living with FH expressed that both their own and their clinician’s willingness to commence treatment, consider additional therapies and understand treatment goals made a difference in improving their cholesterol. A desire for more therapeutic options with fewer side effects was also discussed. However, some did not wish to begin treatment until they fully understood their diagnosis.

## Tailoring Implementation Strategies

Once a needs assessment has been conducted, theory- or evidence-based implementation strategies are selected to address the previously identified barriers and enablers. Implementation strategies can focus on individual and team levels (addressing attitudes, knowledge and skills) or at the organisational level (institutional infrastructure, leadership commitment to change). Taxonomies have been developed to ensure consistent terminology when referring to implementation strategies, like audit and feedback or informing local opinion leaders. The Expert Recommendations for Implementing Change (ERIC) provides a common list of terms and definitions developed by stakeholders with expertise in implementation science and clinical practice [44]. Implementation strategies need to be operationalised to meet local requirements. Specifying who enacts the strategy, the actions or steps that need to be taken, the targets or outcomes of those actions, when the strategy is to be used and at what level of dosage or intensity enables practical application [61].

### Implementation Strategies for FH Detection

Van den Nieuwenhoff et al. [62] report on the impact of family communication strategies in the Netherlands from their population screening program. From 20 semi-structured interviews, the first individual in the family identified often notified first-degree relatives but not more distant relatives, and the conversations with these relatives included stressing the severity of the condition and the threat that inherited high cholesterol poses to their relatives [62].

Findings from the IMPACT-FH Study conducted in the USA (Identification Methods, Patient Activation and Cascade Testing for FH) report on implementation outcomes, guidance on effective messaging and optimization of implementation strategies focused on improving the detection of FH [63]. Jones et al. [64] conducted 5 focus groups with 42 participants, guided by the conceptual model of implementation research, that found the use of automated approaches to identify individuals with FH through the EMR and family communication methods including chatbots and direct contact was acceptable, appropriate and feasible methods to detect FH. Through 11 dyadic interviews and 98 survey respondents’, guidance of effective messaging to motivate cascade testing uptake for FH were suggested and include participants prioritizing messages from four key constructs (severity, susceptibility, response efficacy and self-efficacy), and clinicians could use these constructs to communicate to at-risk relatives about the importance of pursuing diagnosis via cascade testing and subsequent medication management approaches [65]. A forthcoming Campbell et al. study reports on the optimization of these implementation strategies, which is currently undergoing peer review.

### Implementation Strategies for FH Management

Multiple studies have been published from a project aimed to develop implementation strategies to improve treatment approaches for FH described by Jones et al. [40]. This research team conducted a systematic review and meta-analysis of current studies that aimed to improve statin utilisation in individuals with hypercholesterolaemia and mapped methods used to implementation strategies [66]. The results of the systematic review were significantly reduced LDL-cholesterol, increased rates of statin prescribing and improved statin adherence in the implementation strategies; however, not one strategy or group of strategies was associated with these outcomes [66]. But, when at least three strategies were used, it was associated with improvement in LDL-cholesterol levels [66]. Jones et al. [60] developed solutions, or implementation strategies, from qualitative research to address FH treatment. Some suggested solutions included patient and clinician education, transparency of data to the patient, peer groups and clinician champions [60]. One of these solutions, the creation of a new clinical team, creation of a multidisciplinary lipid clinic, was piloted and a program evaluation of its first-year post-implementation found improved diagnosis of FH, increased prescribing of evidence-based therapies and significant reductions in lipid levels [41].

Another research team in the UK has also been investigating implementation strategies to improve FH management. In a qualitative evidence review, they found seven enablers and six barriers to treatment adherence for FH [67]. Kinnear et al. [68] have used the behavioural change wheel and the theoretical domain framework to develop implementation strategies to improve factors related to diet and physical activity treatment guidelines.

## Monitor, Evaluate and Sustain Improvements in Care

Similar to clinical research and quality improvement, it is imperative to evaluate and ensure implementation of guideline recommendations into practice is sustained over time. Monitoring and evaluating implementation efforts requires a focus on the processes required to introduce new evidence into practice. For example, audit and feedback have been demonstrated as an effective implementation strategy across several clinical areas, which could be deployed to change practice in FH [69]. This differs conceptually from evaluating clinical interventions on patient outcomes.

Several FH clinical registries have been established worldwide to collect information for research and health policy planning [70]. Gaps in the care of FH have been well established from analyses of extant registry data [18, 71–76]; the next step is to establish the best approaches to reducing these gaps for different local contexts and scale up these benefits to other sites. This registry infrastructure could be utilised to audit the outcomes of implementation strategies across different jurisdictions to determine whether changes have been successfully sustained over time.

Randomised controlled trials are frequently used in implementation science to evaluate the success of implementation strategies to support a given intervention or program [77, 78]. Given implementation trials must be conducted in real-world settings; innovative designs such as stepped wedge [79, 80], counterbalanced [81, 82] and SMART (sequential multiple assignment randomised trials) or adaptive intervention designs [83] have been developed or redeployed from other fields to rigorously study the process of implementation. Stepped wedge trials stagger the implementation over time to resemble traditional incomplete block designs. Counterbalanced trials allocate units (participants or clusters) to alternative clinical interventions, each also receiving different levels or types of implementation support, so that each intervention-implementation combination balances the other and reduces the risk of study group contamination. SMART or adaptive trials assign participants multiple times sequentially to form a structure for switching or modifying clinical interventions or implementation strategies at specified time points, if beneficial outcomes are not being observed.

## Improving the Translation of Clinical Practice Guidelines with Implementation Science

In summary, implementation science is the study of methods to promote the uptake of research findings into routine healthcare in clinical, organisational or policy contexts [84]. In isolation, the dissemination of clinical practice guidelines is insufficient to implement recommendations into practice. The complexity of health care systems presents many challenges to changing clinical practice, such as ingrained professional and organisational cultures, which must be addressed across multiple levels (individuals, teams, organisations) [85••]. Implementation science offers a raft of methods to understand the nature of changes required to adopt guideline recommendations, categorise the barriers and facilitators to change, design implementation strategies to address challenges and monitor and evaluate benefits and unintended consequences [86, 87].

There is emerging evidence demonstrating that implementation strategies can improve adherence to clinical practice guidelines, as championed by groups such as Cochrane’s Effective Practice and Organisation of Care [88]. In health care, implementation strategies are defined as the specific methods for adopting and sustaining evidence-based programs or interventions [89]. Implementation strategies deemed successful in one setting will usually need to be tailored for another due to differences in contextual circumstances [90]. For example, strategies for initiating FH treatment and target goals have been developed in some settings; however, different healthcare systems must create local models of care and implementation strategies to better recognise and treat FH [27]. It is important to ensure sufficient details are documented to replicate implementation strategies utilised for evidence-based interventions or new models of care [61].

In Europe, there are substantial efforts underway to improve the unacceptably low rates of FH detection by introducing screening in childhood [91, 92]. Implementing the optimal approach to FH screening will depend on the characteristics of individual health systems (e.g. existence of risk reduction pathways and programs, access to diagnostic tests and regulatory frameworks) [93]. However, it appears that universal screening of children and adolescents to identify index cases paired with family cascade testing among relatives of the index case with confirmed FH enables treatment to be initiated at the earliest and most beneficial time, ideally before experiencing a cardiovascular event [93–95]. Importantly, it has been recommended that governments should provide financial support for screening and cascade testing programs and related care, and that implementation research should be conducted to optimise outcomes from these programs and optimised care [96].

**Fig. 1:**
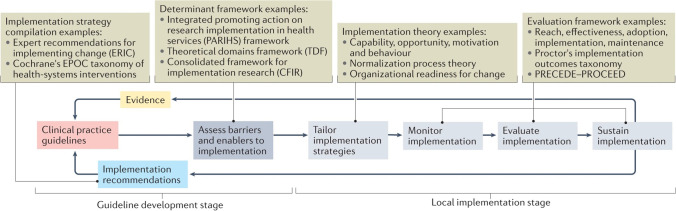
Embedding implementation science into the guideline development and translation processes (source: Sarkies and Jones et al. [85••]. Use of this image is supported by Springer Nature Rights and Permissions)

Recent cardiovascular guidelines have considered implementation and the organisation and development of care, such as the 2018 American Heart Association, American College of Cardiology Cholesterol and Multisociety Guidelines [97] and Integrated Guidance for Enhancing the Care of Familial Hypercholesterolaemia in Australia [5•]. Sarkies et al. [85••] have proposed a model for how engaging implementation scientists in the guideline development process can improve the translation into practice (Fig. 1). This is based on the premise that guideline recommendations should specify both *what* care should be delivered and *how* best to operationalise its delivery. The model incorporates the development of clinical recommendations as well as implementation recommendations for how to organise and deliver care. The model sets out several stages for local adoption: develop clinical and implementation recommendations; assess local barriers and enablers to implementation; tailor implementation strategies; monitor, evaluate and sustain improvements in care.

## Conclusions

Implementing clinical practice guidelines into practice does not occur without active and sustained efforts. Implementation science offers a structured field of research explicitly focussed on achieving improvements in clinical practice for FH. Many factors influence research translation, such as professional and organisational cultures, resource constraints and computability with existing workflows, which can create resistance to health system reform. Implementation science frameworks have been used to overcome this system inertia. Analysis of clinical registries and clinician surveys have identified evidence to practice gaps that could represent high priorities for the implementation of guideline recommendations into practice. The barriers and enablers to overcoming these gaps in care for FH detection and management represent ideal targets for implementation strategies, and we provide several example studies internationally which have applied tailored strategies to improve FH care. Monitoring, evaluating and sustaining these improvements long-term is needed to refine implementation strategies and enable them to be generalised across different jurisdictions. Future research is needed to determine the effectiveness and cost-effectiveness of specific implementation strategies for improving aspects of FH care. Clinician training in implementation science, engaging patient advocates and other key stakeholders and embedding implementation processes into the development of clinical practice guidelines is recommended.
